# Machine learning-based prediction of post-stroke cognitive status using electroencephalography-derived brain network attributes

**DOI:** 10.3389/fnagi.2023.1238274

**Published:** 2023-09-28

**Authors:** Minwoo Lee, Yuseong Hong, Sungsik An, Ukeob Park, Jaekang Shin, Jeongjae Lee, Mi Sun Oh, Byung-Chul Lee, Kyung-Ho Yu, Jae-Sung Lim, Seung Wan Kang

**Affiliations:** ^1^Department of Neurology, Hallym University Sacred Heart Hospital, Hallym Neurological Institute, Hallym University College of Medicine, Anyang, Republic of Korea; ^2^iMedisync, Inc., Seoul, Republic of Korea; ^3^Department of Neurology, Hwahong Hospital, Suwon, Republic of Korea; ^4^Department of Neurology, Asan Medical Center, University of Ulsan College of Medicine, Seoul, Republic of Korea

**Keywords:** ischemic stroke, cognition, electroencephalography, functional network, machine learning

## Abstract

**Objectives:**

More than half of patients with acute ischemic stroke develop post-stroke cognitive impairment (PSCI), a significant barrier to future neurological recovery. Thus, predicting cognitive trajectories post-AIS is crucial. Our primary objective is to determine whether brain network properties from electroencephalography (EEG) can predict post-stroke cognitive function using machine learning approach.

**Methods:**

We enrolled consecutive stroke patients who underwent both EEG during the acute stroke phase and cognitive assessments 3 months post-stroke. We preprocessed acute stroke EEG data to eliminate low-quality epochs, then performed independent component analysis and quantified network characteristics using iSyncBrain®. Cognitive function was evaluated using the Montreal cognitive assessment (MoCA). We initially categorized participants based on the lateralization of their lesions and then developed machine learning models to predict cognitive status in the left and right hemisphere lesion groups.

**Results:**

Eighty-seven patients were included, and the accuracy of lesion laterality prediction using EEG attributes was 97.0%. In the left hemispheric lesion group, the network attributes of the theta band were significantly correlated with MoCA scores, and higher global efficiency, clustering coefficient, and lower characteristic path length were associated with higher MoCA scores. Most features related to cognitive scores were selected from the frontal lobe. The predictive powers (*R*-squared) were 0.76 and 0.65 for the left and right stroke groups, respectively.

**Conclusion:**

Estimating EEG-based network properties in the acute phase of ischemic stroke through a machine learning model has a potential to predict cognitive outcomes after ischemic stroke.

## Introduction

Stroke is the major cause of mortality and disability worldwide (Wolfe, 2000; Langhorne et al., 2011). Among disabilities, cognitive impairment after stroke exerts an important effect on patients, functional recovery, and long-term prognosis. Currently, clinicians evaluate the risk of post-stroke cognitive impairment using structural information, such as the location and size of the stroke lesion, from magnetic resonance imaging (MRI) or computed tomography (CT). An international large-scale consortium for lesion-symptom mapping research recently presented important findings on the location in the brain of lesions that cause cognitive impairment following stroke (Weaver et al., 2021b). However, these methods using structural brain imaging are based on the premise that brain function is selectively impaired in the localized area where the stroke lesion occurred. However, in practice, lesions at several sites might result in the same symptoms and signs, which is accounted for by the disruption of a shared neural network (Fox, 2018). As a result, it would be difficult to predict cognitive impairment after stroke based only on lesion location as identified in MRI, and it is necessary to evaluate the widespread effect of a lesion on the entire brain (Stinear, 2010; Carter et al., 2012). Tools for measuring functional connectivity, which evaluates not only a specific brain region’s activity but also the interaction between different regions, may be essential in resolving this issue (Bressler and Menon, 2010; Stinear, 2010; Aerts et al., 2016).

Among the available modalities to explore the functional activity of the human brain, disconnectome studies using functional MRI (fMRI) and diffusion tensor imaging(DTI) are being actively investigated in post-stroke cognitive impairment studies (Biesbroek et al., 2021; Lim et al., 2021). However, fMRI and DTI are difficult to apply in stroke patients in clinical practice due to their high cost and vulnerability to motion artifacts. As an alternative, electroencephalography (EEG) is advantageous, as EEG can detect functional changes in the entire brain caused by lesions, and it is less expensive than MRI (Chen et al., 2013). Though the low spatial resolution of EEG is caused by the limited number of electrodes and by the effect of the electromagnetic field on volume conduction, several methods have been proposed to overcome these shortcomings. Standardized low-resolution brain electromagnetic tomography (sLORETA) is a mathematical tool for source localization to map real brain regions from recorded EEG signals (Jatoi et al., 2014). Imaginary coherence (iCOH) calculated from the imaginary part of coherence (Sanchez Bornot et al., 2018) also helps to overcome the low spatial resolution of EEG even though iCOH is not a direct source localization method. It can be used to determine the functional connectivity between two regions of interest (ROIs) and efficiently reduce volume conduction (Nolte et al., 2004).

Several studies have reported that quantitative analysis of EEG may be employed to predict prognosis after acute ischemic stroke. Specific frequency bands, power ratios between different frequency bands, and symmetric index were strongly correlated with patients’ stroke severity index using the National Institutes of Health Stroke Scale (NIHSS) and with the functional status assessed by the modified Rankin Scale (Sheorajpanday et al., 2011; Finnigan and van Putten, 2013). Further, several QEEG indices, such as relative theta frequency (Schleiger et al., 2017) or irregularity of spectral power of frontal lobes (Hadiyoso et al., 2022), predicted post-stroke cognitive impairment in the previous studies with limited explanatory power. As incorporating brain network attributes may provide more details about cognitive prognosis after stroke, QEEG using a machine learning approach to incorporate functional connectivity along with lesion characteristics would be a powerful tool that could reveal the network vulnerability for post-stroke cognitive impairment in stroke patients.

Therefore, we aimed to investigate which EEG-dervied brain networks are associated with short-term cognitive status after stroke and to assess whether the brain network can accurately predict cognitive status after acute ischemic stroke using a machine learning approach.

## Methods

### Study population

Of the 1,959 consecutive ischemic stroke patients admitted between September 2016 and February 2020, 1,240 patients had an anterior circulation stroke and were admitted to the hospital within 7 days of symptom onset. Out of these, 223 underwent a 3-month MoCA. After excluding 18 patients with a premorbid cognitive impairment, as indicated by an Informant Questionnaire on Cognitive Decline in the Elderly (IQCODE) score (Lee et al., 2005) of ≥3.6, a total of 87 patients who had an EEG during the acute stroke phase were selected for our study.

This study was approved by the Institutional Review Board of the Hallym University Sacred Heart Hospital and was not required to seek additional consent because of its retrospective nature and due to the minimal risk to participants.

### Neuropsychological and clinical variables

The participants underwent neuropsychological tests, including the Korean version of the Mini-Mental State Examination (MMSE) and the Korean version of the Montreal Cognitive Assessment (MoCA) during admission (baseline) and after 3 months. A trained neuropsychologist performed the tests. Standardized scores for each test were calculated based on age, sex, and education-adjusted norm (Kang et al., 2009). Change values were calculated as “3-month test score–baseline test score.

From a stroke registry database (Bae et al., 2022),demographic factors, including age, sex, and years of education, were collected. Vascular risk factors, including history of hypertension, diabetes, hyperlipidemia, atrial fibrillation, smoking status, coronary heart disease, and previous stroke or transient ischemic attack, were also collected. The evaluation of stroke characteristics involved the assessment of initial stroke severity based on the NIHSS score and stroke subtypes according to the Trial of ORG 10172 in Acute Stroke Treatment (TOAST) classification. Premorbid functional status was evaluated using the modified Rankin scale. Time intervals between index stroke, EEG, and neuropsychological evaluations (baseline and follow-up) were also determined.

### Brain imaging

Neuroimaging analysis was performed using an MRI acquired at the time of the stroke for medical purposes. The participants underwent 3.0-Tesla MRI scanning (Achieva, Philips Healthcare, Eindhoven, The Netherlands). The protocols consisted of fluid-attenuated inversion recovery imaging (FLAIR), axial T1- and T2-weighted spin echo, gradient-echo imaging, coronal T1-weighted spin echo imaging, and diffusion-weighted imaging (DWI). As for acute lesions, multiplicity, left hemisphere involvement, and cortical involvement of stroke lesions were investigated. As for chronic lesions, white matter hyperintensities (Fazekas scale), lacunes and cerebral microbleeds (presence and count), and medial temporal lobe atrophy (Scheltens visual scale) (Scheltens et al., 1992) were evaluated using the STRIVE criteria (Wardlaw et al., 2013).

### EEG data acquisition

We retrospectively collected EEG data taken within 2 weeks of onset in patients with acute ischemic stroke. The EEG was recorded at rest using 19 channels of the international 10–20 system according to the laboratory’s internal standardized guidelines. To enhance the data quality and mitigate artifact effects, the EEG data underwent preprocessing. In the initial stage, the signals were sampled at a rate of 250 Hz and subjected to bandpass filtering in the frequency range of 1 ~ 45.5 Hz. Further, notch filtering was applied to eliminate unwanted noise. Subsequent steps involved re-referencing using common average referencing (CAR), the rejection of bad epochs through artifact subspace reconstruction (ASR), and the removal of stationary noise using adaptive mixture independent component analysis (AMICA) (Mullen et al., 2015; Blum et al., 2019) in iSyncBrain®. Additionally, artifacts attributed to electromyogram (EMG) and electrooculogram (EOG) were eliminated to generate reliable QEEG data. All preprocessing steps, including sensor-level data handling and source-level data computation and extraction, were performed using the cloud-based EEG analysis platform, iSyncBrain® provided by iMediSync, Inc. Korea^1^ (Subasi and Ismail, 2010). Each component was analyzed, and noise components, such as heartbeat and muscle behavior, were excluded in this process. The automatically cleaned data were inspected manually once again to filter out the unfiltered noise and check whether the cleaned data were analyzable or not.

After the signal processing, spectrum powers were measured from 19 channels and 8 frequency bands. The 8 frequency bands were delta (1–4 Hz), theta (4–8 Hz), alpha1 (8–10 Hz), alpha2 (10–12 Hz), beta1 (12–15 Hz), beta2 (15–20 Hz), beta3 (20–30 Hz), and gamma (30–45 Hz). All the power values were also transformed into their relative values, which is the ratio of a specific band power to the entire band power. Also, the power ratios between two spectral bands, namely, TAR (Theta/Alpha), TBR (Theta/Beta), TBR2 (Theta/Beta2), and DAR (Delta/Alpha), were calculated at each channel. The next step was source power estimation. Source cortical activity was mathematically estimated by using sLORETA (Pascual-Marqui, 2002). Subsequently, the spectrum powers were expanded from 19 channels to 68 cortical ROIs based on the Desikan–Killiany atlas (Desikan et al., 2006), providing us with more detail on brain activity and the source of spatial information. Following the Desikan–Killiany atlas, the 68 ROIs are arranged in frontal, temporal, parietal and occipital lobe. Even and odd numbers were used for the ROIs in the left and right hemispheres, respectively (Supplementary Table S1). Based on each source power, the functional connectivity between two ROIs was estimated using the iCOH method, which is calculated as


(1) $$iCOH=Im\left(COH\right)=Im\left(\frac{S_{xy}\left(f\right)}{\sqrt{S_{xx}\left(f\right)S_{yy}\left(f\right)}}\right)$$

where $S_{xy}\left(f\right)$ is the cross-power spectral density of ROI x , y and $S_{xx}\left(f\right)$ , $S_{yy}\left(f\right)$ are the power spectral density of ROI x , y . The iCOH method is robust in volume conduction so that we can separate the brain’s functional connectivity from the electromagnetic field effect. Finally, the network was constructed using each ROI as a node and the iCOH between ROIs as an edge. Four network features (global efficiency, characteristic path length, clustering coefficient, and modularity) (Liu et al., 2017) were calculated based on the iCOH values. Considering that each node had the iCOH values for all other nodes, we retained only the top 25% of the iCOH values and used them to construct a network having only a quarter of the edges being connected. The detailed information and the number of network features used in this study are shown in Supplementary Table S2.

### Prediction of a stroke lesion’s laterality

First, we categorized patients into two groups based on lesion laterality for further analysis to uncover novel predictors independent of lesion laterality, as previous research has indicated that left-sidedness of the infarct site is a robust predictor of cognitive impairment (Weaver et al., 2021a). Based on prior studies showing that slow waves predominate close to the stroke (Sheorajpanday et al., 2011), we trained the machine learning model to classify the laterality of the infarct regions and used the classified results for the next step. According to the predictions of the machine learning method built using data from patients with clearly left- or right-sided lesions, individuals with both hemispheric lesions were divided into left or right laterality.

### Prediction of post-stroke cognitive status

**Figure 1:**
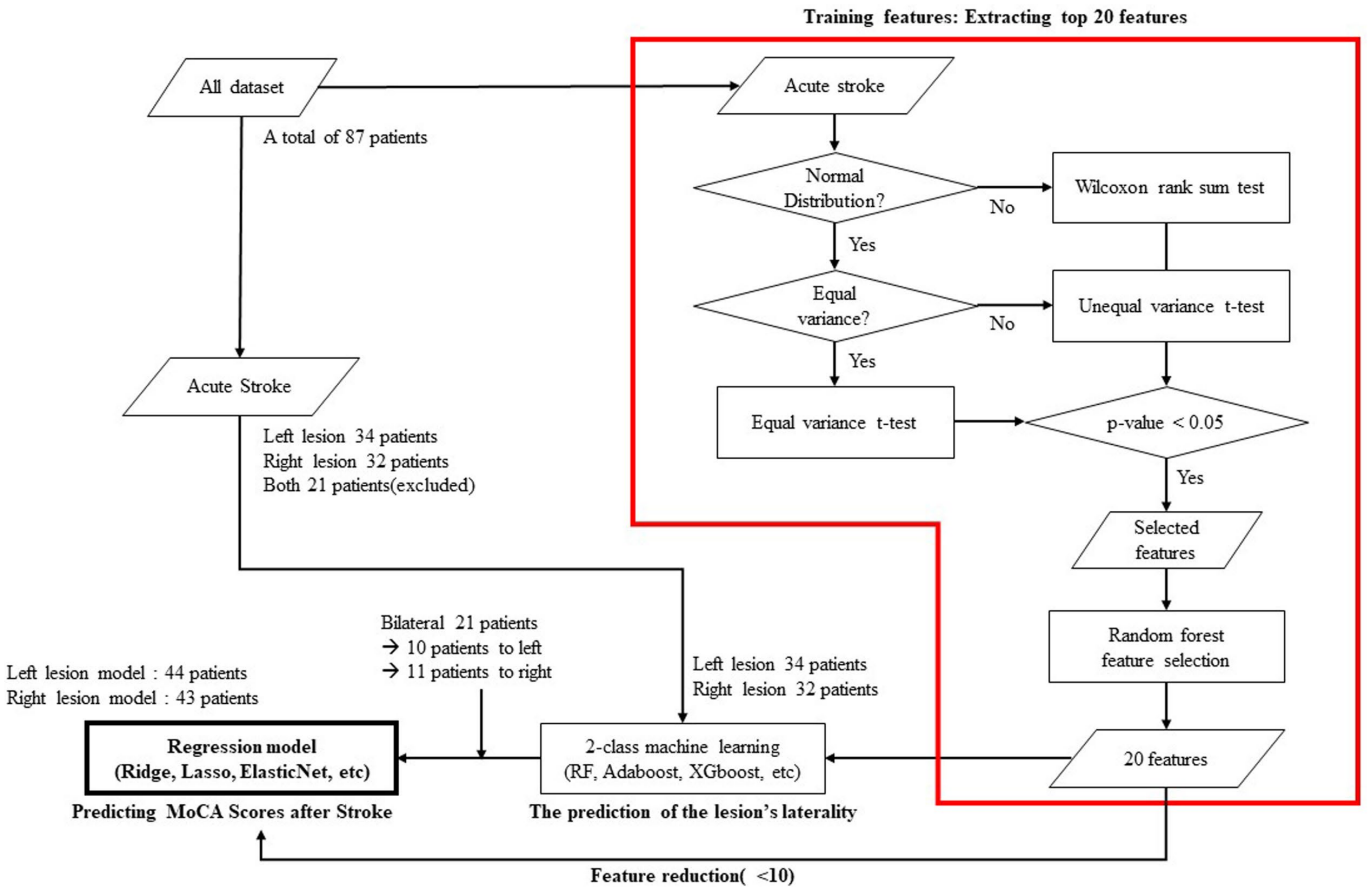
Flow chart showing the statistical verifications, feature selection, and machine learning employed to predict cognitive status 3-month after stroke.

For each of the two groups dichotomized by the laterality of the lesion, we constructed a regression model with cognitive level as the outcome variable. Among various EEG features, including spectral power, power ratio, source power, connectivity, and network measures, we only utilized network variables as explanatory variables because our hypothesis was to predict cognitive prognosis from functional network characteristics and to reduce the possibility of overfitting by limiting the number of explanatory variables. We first calculated the correlation coefficient between the MoCA standardized score and each network feature extracted from the EEG. For further analysis, we extracted only those network features with a correlation coefficient higher than 0.3. The extracted features were arranged in their order of importance as calculated using a machine learning model. The importance means how much each feature is used to predict the output variable in each node. It is calculated by the ratio between the importance of each feature to the summation of all the importance. In this model, the top 20 features were selected. In the final process, machine learning-based regression models for post-stroke MoCA standardized scores were generated using Ridge, Lasso, ElasticNet, support vector regression, and AdaBoost regression. The flow chart of the model including the prediction of the lesion’s laterality is shown in Figure 1 and each model’s explanatory power was calculated by *R*-squared as follows:


(2) $$R^{2}=1-\frac{SS_{res}}{SS_{tot}}=1-\frac{\sum {\left(y_{i}-{\hat{y}}_{i}\right)}^{2}}{\sum {\left(y_{i}-\bar{y}\right)}^{2}}$$

where $SS_{res}$ is the residual sum of squares, $SS_{tot}$ is the total sum of squares, $y_{i}$ is the actual value, ${\hat{y}}_{i}$ is the predicted value, and $\bar{y}$ is the mean value of the total actual values.

All statistical analyses were performed using R version 4.0.5, and statistical significance was set at *p* < 0.05.

### Leave-one-out (LOO) validation technique

For the validation of our machine learning models, we employed the Leave-one-out (LOO) method. This approach is especially suited for limited datasets. Our dataset comprises data from 87 patients, and given the study’s objective, separate models were developed for the left and right brain regions, further restricting the available training data. The LOO technique, a variant of k-fold cross-validation, involves using each data point in the dataset for both training and testing (Cawley and Talbot, 2004). Given the limited size of our training data, this method allows for an in-depth sensitivity analysis on individual data points, offering insights into the model’s responsiveness to specific observations.

### Sensitivity analysis

To bolster the robustness of our findings, we undertook additional machine learning analyses with a focus on minimizing potential overfitting by adjusting the number of incorporated features. Our approach involved: (1) Prioritizing features based on their effect size importance and (2) integrating alpha values from the top 5 features with beta values sourced from the bottom 15 features. Models were then selected based on their *R*-squared values. By employing a comprehensive strategy, which included the selection of 3 to 5 features from the top quintile and varying numbers from the bottom 15 (with a cumulative count not exceeding 10 features), we were able to discern models that exhibited the optimal *R*-squared values.

## Results

### Baseline characteristics and stroke features of the study population

A total of 87 patients with acute stroke who underwent MRI, EEG, and the MoCA were enrolled in this study. The mean age of the patients was 65.4 years (SD 11.6), and 54 (61.4%) of them were men. The median scores for NIHSS and educational attainment were 3 (IQR 1–6) and 9.5 years (IQR 6–12), respectively (Table 1). The time intervals from stroke onset to the EEG and cognitive tests had a median of 4 days (IQR 2–6) and 97 days (IQR 90.0–103.0), respectively. The 3-month MoCA score had a median of 23 points (IQR 19–26).

**Table 1:** Baseline characteristics of the study population.

	*N* = 87
*Demographics*	
Age	65.4 ± 11.7
Male	53 (60.9)
Education, years	10 [6–12]
*Vascular risk factors*	
Hypertension	51 (58.6)
Diabetes mellitus	25 (28.7)
Hyperlipidemia	23 (26.4)
Smoking	30 (34.5)
Atrial fibrillation	14 (16.1)
Coronary heart disease	7 (8.1)
Previous stroke/transient ischemic attack	15 (17.2)
*Index-stroke characteristics*	
Premorbid modified Rankin scale >0	3 (3.4%)
TOAST	
LAA	32 (36.8)
SVO	24 (27.6)
CE	13 (14.9)
Other-determined	2 (2.3)
Undetermined	16 (18.4)
Initial NIHSS	3.0 [1.0–6.0]
*Acute lesions characteristics*	
Multiple lesions	41 (48.8)
Left hemispheric lesions	51 (60.7)
Cortical lesions	37 (44.1)
Chronic lesions	
Medial temporal lobe atrophy	
0 / 1 / 2 / 3 / 4	10 (12.1) / 39 (47.0) / 29 (35.0) / 4 (4.8) / 1 (1.2)
PVWMH	
1 / 2 / 3	58 (69.1) / 17 (20.2) / 9 (10.7)
SCWMH	
1 / 2 / 3	56 (66.7) / 19 (22.6) / 9 (10.7)
Lacunes	29 (34.5)
Number of lacunes	0 [0–1]
CMB	31 (36.5)
Number of CMBs	0 [0–1]
*Time intervals*	
Index-stroke to EEG	3 [2–5]
Index-stroke to baseline neuropsychological evaluations	4 [2–6]
Index-stroke to follow-up neuropsychological evaluations	97 [90–103]

Continuous variables were presented as mean ± standard deviation for normally distributed data or as median with IQR for skewed data; categorical variables were presented as frequencies (proportions). TOAST (Trial of ORG 10172 in Acute Stroke Treatment), LAA (large-artery atherosclerosis), SVO (small vessel occlusion), CE (cardioembolism), NIHSS (National Institutes of Health Stroke Scale), PVWMH (periventricular white matter hyperintensities), SCWMH (subcortical white matter hyperintensities), CMB (cerebral microbleeds), EEG (electroencephalography).

### Lesion laterality prediction

During the prediction of the lesion laterality process, 21 patients were excluded because they had infarct regions in both hemispheres. Thus, they were not labeled or used to train the laterality prediction model. The model predicting lesion laterality showed high accuracy, sensitivity, and specificity at 96.97, 97.01, and 96.88%, respectively. The 21 patients excluded during the training due to bilateral lesions were included according to the predicted lesion sides. More specifically, ten patients were classified into the left lesion group, and the remaining 11 patients were in the right lesion group. Finally, 44 patients were categorized into the left and 43 patients into the right lesion group. The model performance according to the number of features from 5 to 10 are demonstrated in the Supplementary Table S3.

### Distribution of the selected features over the frequency band and ROIs, and summation of feature importance

The distribution of the selected features over the frequency band and the summation of their feature importance as calculated from the feature selection model are shown in Table 2. Theta, alpha2, and beta1-related features were chosen as significant explanatory factors in the model for the left stroke group, but only theta-related features were chosen for the right stroke group. Regarding feature importance, theta and beta1 exhibited the most significant values in the models for the left and right stroke groups, respectively. For the cortical region distribution, the left and right stroke models were similar in selecting most features from the frontal lobe. The number of important features from the frontal lobe was 65% in both models. Specifically, 13 and 11 features were located in the ROI 1–20 for the left and right models, respectively. The summation of feature importance of the features located in the frontal lobe was 0.50 and 0.46 for the left and right models, respectively, which is 73.47 and 68.06% of the total importance.

**Table 2:** Distribution of the selected features over the frequency band and ROIs, and summation of feature importance.

	Number of features		Summation of feature importance	
	Left stroke group	Right stroke group	Left stroke group	Right stroke group
*Frequency bands*				
Delta	1 (5%)	2 (10%)	0.04 (14.09%)	0.05 (8.53%)
Theta	10 (50%)	1 (5%)	0.05 (17.54%)	0.03 (4.74%)
Alpha1	1 (5%)	2 (10%)	0.02 (6.15%)	0.04 (7.00%)
Alpha2	5 (25%)	4 (20%)	0.11 (42.43%)	0.17 (27.94%)
Beta1	2 (10%)	4 (20%)	0.03 (12.76%)	0.13 (21.71%)
Beta2	1 (5%)	6 (30%)	0.02 (7.03%)	0.16 (25.36%)
Beta3	0 (0%)	0 (0%)	0 (0%)	0 (0%)
Gamma	0 (0%)	1 (5%)	0 (0%)	0.03 (4.71%)
Total	20 (100%)	20 (100%)	0.26 (100%)	0.61 (100%)
*Region of interests*				
Frontal lobe	13 (65%)	13 (65%)	0.50 (73.47%)	0.46 (68.06%)
Temporal lobe	4 (20%)	2 (10%)	0.10 (15.48%)	0.04 (5.32%)
Parietal lobe	1 (5%)	1 (5%)	0.03 (4.12%)	0.02 (3.54%)
Occipital lobe	1 (5%)	3 (15%)	0.02 (3.60%)	0.07 (9.79%)
Isthmus (excluded from 4 lobes)	1 (5%)	0 (0%)	0.02 (3.33%)	0 (0%)
Others	0 (0%)	1 (5%)	0 (0%)	0.03 (3.87%)
Total	20 (100%)	20 (100%)	0.6751 (100%)	0.61 (100%)

### EEG-based network features and machine learning models for predicting MoCA scores after stroke

**Figure 2:**
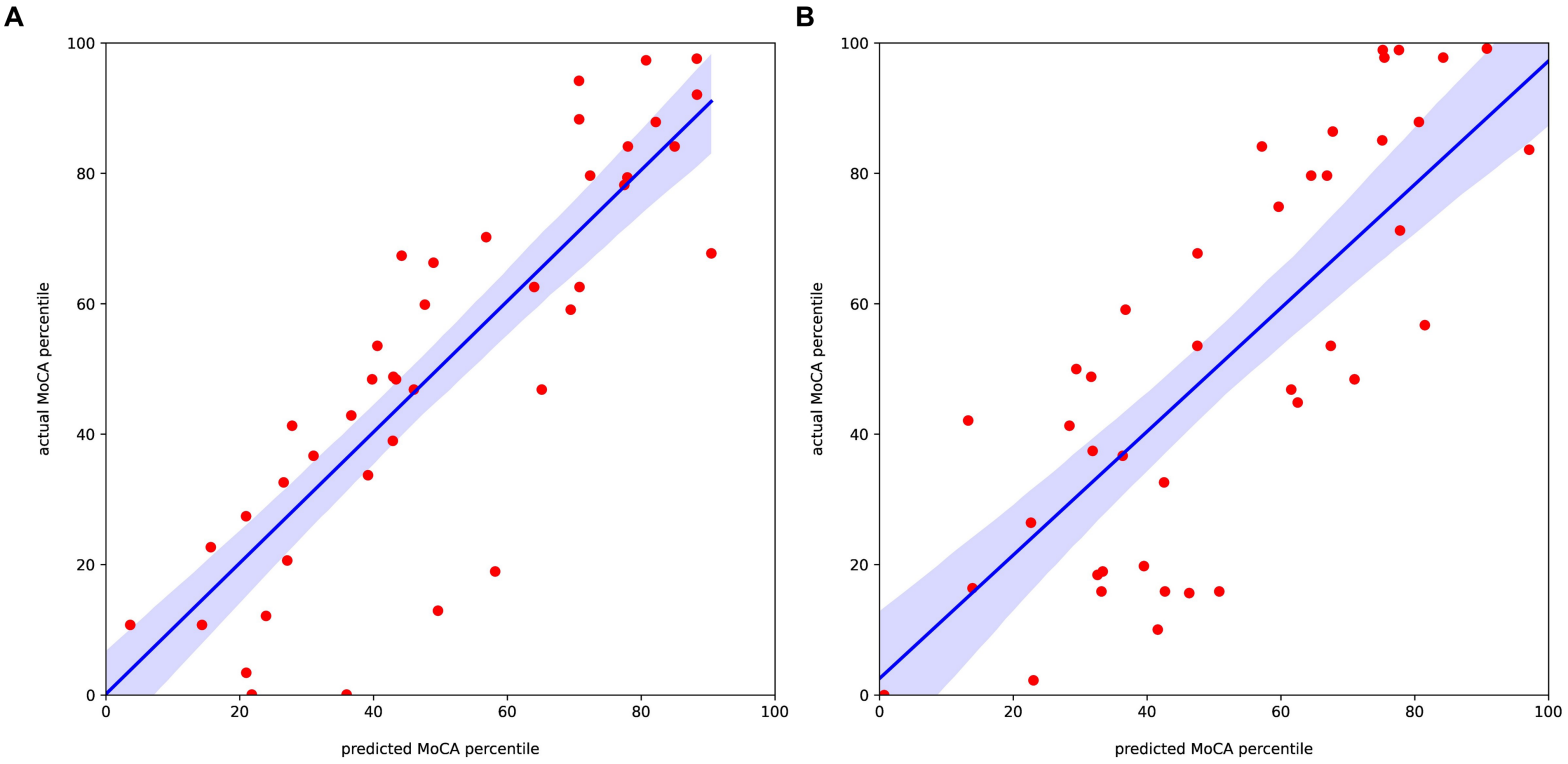
Regression results predicting the MoCA percentile score relative to the actual value for the **(A)** left and **(B)** right stroke estimated group.

The correlation between the 20 selected features and the standardized scores of the MoCA after 3 months for the left and right stroke groups are demonstrated in Supplementary Tables S4, S5, respectively. In the case of the left stroke group, all features related to the theta band showed a positive correlation coefficient with the MoCA standardized scores. By contrast, the correlation in the right stroke group does not show a clear relationship between the selected features and the target value. Using the 20 selected features, we trained the aforementioned machine learning regression models, and the results are shown in Figure 2 and Table 3. As shown in Table 3, Ridge regression demonstrated the best performance in both groups (*R*-squared 0.76 for the left stroke group, 0.65 for the right stroke group). Our model did not incorporate patients’ clinical variables that were not derived from the EEG data. Nonetheless, certain variables, like age and baseline MoCA scores, could potentially influence the MoCA score at 3 months. As part of a sensitivity analysis, we also evaluated the model’s performance when it included age, baseline MoCA scores, and baseline NIHSS scores. However, this addition did not enhance the model’s predictive capability, with *R*-squared values of 0.6174 and 0.6207 for the left and right stroke groups, respectively.

**Table 3:** *R*-squared results of the machine learning regression models.

Network type	Network features only (left/right)	All EEG features (left/right)
Ridge	0.76/0.65	0.64/0.48
Lasso	0.71/0.57	0.61/0.35
ElasticNet	0.63/0.55	0.44/0.37
Support Vector Regression	0.17/0.26	0.07/0.01
AdaBoost Regression	0.54/0.54	0.62/0.63

### Sensitivity analysis with reduced variable count

In our sensitivity analyses with a reduced number of variables, the left lesion model, incorporating the top 8 features, achieved an *R*-squared value of 0.57. Conversely, the right lesion model, leveraging the top 10 features, reached an *R*-squared value of 0.51 (refer to Supplementary Figure S1). We recognized that simplifying features based purely on their significance could potentially diminish the model’s explanatory capability. To circumvent this, we crafted a composite model, opting for 3 to 5 of the top 5 features, and complementing with a selection from the bottom 15, ensuring the total did not surpass 10 features. This intricate procedure entailed evaluating close to 9,000 models to pinpoint those with the most robust *R*-squared values.

As evidenced in the Supplementary Table S6, three models from the left lesion exceeded the performance of the top 20 feature-oriented models in terms of their explanatory capacity. In contrast, for the right lesion, a singular model outperformed the foundational model. The superior model for the left lesion amalgamated the top 5 features with an added 5 from the lower end, registering an *R*-squared value of 0.77. As for the right lesion, the eminent model incorporated the top 3 features and a supplementary 7 from the bottom tier. The definitive variables chosen by these exemplary models, along with the regression plots, are detailed in Supplementary Figures S2, S3.

## Discussion

In our study, the brain network attributes, which were analyzed using the EEG data obtained at the acute stage after stroke through machine learning, were useful in predicting the MoCA score at 3 months after acute stroke. Furthermore, this prediction was made based only on EEG analysis results without considering other clinical information, and this approach may be useful in clinical practice.

As expected, the features selected in the machine learning models showed different trends between the left and right stroke groups. In the patients with the left hemispheric lesion group, we identified many significant features related to the theta band, whereas the right lesion group had only a few features distributed in the theta band. In the left hemispheric lesion group, the network attributes in the theta band were consistently and significantly correlated with the post-stroke MoCA scores at 3 months after stroke. The higher the global efficiency or clustering coefficient, or the lower the characteristic path length, the higher the MoCA score. However, the directionality of this association was not observed in the right hemispheric lesion group.

Network properties can be categorized in terms of integration (global efficiency and characteristic path lengths) and segregation (clustering coefficients and modularity) to explore their association with post-stroke cognitive impairment (Lim et al., 2021). In a previous study, a gradual recovery of reduced modularity after an acute stroke to the level of the control group was associated with improvements in memory and attention scores (Siegel et al., 2018). Another study showed the association between reduced global efficiency and post-stroke depression (Xu et al., 2019). In our results, the global efficiency of the theta band was the most important explanatory factor in the left stroke group, and the clustering coefficient of the alpha band was found to be important in the right stroke group (Siegel et al., 2018). Contrary to the findings of a previous study, our results did not underscore the significance of modularity. This discrepancy may be attributable to the smaller quantity of modularity features included in our analysis compared to other features. As previously discussed, modularity shares conceptual similarities with the clustering coefficient, and our analysis revealed that the clustering coefficient constituted the majority of the top 20 selected features, irrespective of lesion laterality.

The top 20 important EEG network features reflecting cognitive prognosis were also more often extracted from the opposite side of the lesion. In the case of the left stroke group, 12 features were selected from the right hemisphere, and 8 were selected from the left hemisphere. In the case of the right stroke group, 12 features were selected from the left hemisphere, and 7 features were selected from the right hemisphere. Previous studies have shown that contralesional hemispheric networks were activated for compensatory processes during functional recovery after stroke (Rehme et al., 2011). During subacute periods after stroke, a positive correlation of contralesional, homologous regions with ipsilesional regions exerts a beneficial effect on muscle strength recovery (Rehme et al., 2011). However, these correlations were dependent on time since the stroke, and our results show that some features of the contralateral hemisphere are negatively correlated with cognition, so further research is needed. Prior to the prediction, we divided the dataset into two subsets based on the laterality of stroke infarct, as it affects the overall EEG signal. Then, we selected the 20 most important features from the network variables estimated from functional connectivity using the iCOH between 68 ROIs. The selected features almost belonged to specific frequency bands, namely, theta, fast alpha, and slow beta, even though the left and right stroke estimated groups showed different tendencies. Several studies also proposed the relationship between post-stroke cognitive impairment and these frequency bands (De Vico et al., 2009; Al-Qazzaz et al., 2018; Doerrfuss et al., 2019). In the case of the ROIs of the network features, both the left and right stroke groups showed consistent results, wherein most of the features were concentrated in the frontal lobe, especially in the ROI 1–20 (Schleiger et al., 2014). It is known that frontal executive function, attention, and processing speed are mainly impaired in stroke patients (Lo et al., 2019), and MoCA is reported to be more sensitive in detecting frontal dysfunction than other tests, such as MMSE (Duncan and Owen, 2000; Fuster, 2002; Pendlebury et al., 2012; Braakman et al., 2013; Braun et al., 2015). In this sense, the regression results support the relationship between predictive cognitive status and brain network features, predominantly of frontal origin.

Our primary analysis consisted of only network characteristics as explanatory variables, as hypothesized. We also examined whether explanatory power improved in sensitivity analyses using all functional connectivity features, including iCOH. However, the prediction results were worse than those obtained using network features only. It is not uncommon when certain variables irrelevant to the outcome of interest are included in the machine learning model that eventually adds noises and reduces the accuracy of predictability. Further, in cases with many independent variables, several statistical problems arise due to multiple comparison issues. In this case, reducing dimension is a useful approach to finding biologically relevant results (Lim et al., 2021). The iCOH values offer considerably more information, so they are highly correlated with other iCOH values obtained in the same or adjacent ROIs. By contrast, the network variables of each ROI provide relatively independent information from other ROIs, and it is assumed that the independence between input features provided better results.

Our study has several limitations. Firstly, the sample size of stroke patients was insufficient to establish an independent validation cohort for testing the derived machine learning model. Consequently, we were compelled to implement the leave-one-out technique to enhance the reliability of both the training set and validation outcomes. Secondly, our limited patient population did not allow for examining differences attributable to varying stroke mechanisms. Furthermore, we gauged cognitive function solely through MOCA, excluding comprehensive neuropsychological testing. This restricts our ability to ascertain potential variations in correlations between EEG characteristics and individual cognitive domains. Additional inherent limitation is our choice of threshold for brain network indicators, set at the 1/4 level. While this decision balanced detail and feasibility, the optimal thresholding remains an area for future refinement and validation. Finally, patients who were unable to perform neuropsychological tests were excluded. Consequently, our findings may not be universally applicable to all ischemic stroke patients. Administering cognitive tests to every ischemic stroke patient in a real-world context presents practical challenges. This inherent bias, stemming from attrition, has been extensively discussed in our earlier sections. Readers should exercise caution and keep this context in mind while interpreting our results (Pendlebury et al., 2015).

## Conclusion

The predictive accuracy of post-stroke cognitive function may be enhanced by evaluating network characteristics via a machine learning-based EEG analysis. Therefore, implementing EEG during the acute phase of a stroke could serve as a reliable method for anticipating short-term cognitive prognosis following an ischemic stroke. Further, prediction of post-stroke cognitive status after stroke may present a promising avenue for enhancing the precision medicine for post-stroke care.

## Data availability statement

The raw data supporting the conclusions of this article will be made available by the authors, without undue reservation.

## Ethics statement

The studies involving humans were approved by Institutional Review Board of the Hallym University Sacred Heart Hospital. The studies were conducted in accordance with the local legislation and institutional requirements. The ethics committee/institutional review board waived the requirement of written informed consent for participation from the participants or the participants’ legal guardians/next of kin because of its retrospective nature and due to the minimal risk to participants.

## Author contributions

ML, YH, SA, J-SL, and SK contributed to conception and design of the study. UP organized the database. JS and YH performed the statistical analysis. SA and YH wrote the first draft of the manuscript. JL, MO, B-CL, K-HY, and ML wrote sections of the manuscript. All authors contributed to the article and approved the submitted version.

## Footnotes

^1^ https://isyncbrain.com
