# Supplementary material for: Machine learning-based prediction of post-stroke cognitive status using electroencephalography-derived brain network attributes
Source: Front Aging Neurosci. 2023 Sep 28;15:1238274. doi: 10.3389/fnagi.2023.1238274 (PMC10568623; doi:10.3389/fnagi.2023.1238274)
Supplement: Supplementary file 1 [file Table_1.docx]

**Supplemental Table 1. 68 ROIs of Desikan-Killiany atlas and their positions arranged in 4 lobes**

| ROI number | ROI name | Lobe |
| --- | --- | --- |
| 1/2 | Frontal pole (left/right) | Frontal lobe |
| 3/4 | Superior frontal gyrus (left/right) |  |
| 5/6 | Rostral middle frontal gyrus (left/right) |  |
| 7/8 | Caudal middle frontal gyrus (left/right) |  |
| 9/10 | Pars opercularis inferior frontal gyrus (left/right) |  |
| 11/12 | Pars orbiltalis inferior frontal gyrus (left/right) |  |
| 13/14 | Pars triangularis inferior frontal gyrus (left/right) |  |
| 15/16 | Medial orbitofrontal cortex (left/right) |  |
| 17/18 | Lateral orbitofrontal cortex (left/right) |  |
| 19/20 | Precentral gyrus (left/right) |  |
| 21/22 | Paracentral lobule (left/right) |  |
| 23/24 | Rostral anterior cingulate cortex (left/right) |  |
| 25/26 | Caudal anterior cingulate cortex (left/right) |  |
| 27/28 | Temporal pole (left/right) | Temporal lobe |
| 29/30 | Superior temporal gyrus (left/right) |  |
| 31/32 | Middle temporal gyrus (left/right) |  |
| 33/34 | Inferior temporal gyrus (left/right) |  |
| 35/36 | Transverse temporal cortex (left/right) |  |
| 37/38 | Banks of the superior temporal sulcus (left/right) |  |
| 39/40 | Fusiform gyrus (left/right) |  |
| 41/42 | Entorhinal cortex (left/right) |  |
| 43/44 | Parahippocampal gyrus (left/right) |  |
| 45/46 | Insula (left/right) |  |
| 47/48 | Postcentral gyrus (left/right) | Parietal lobe |
| 49/50 | Superior parietal cortex (left/right) |  |
| 51/52 | Inferior parietal cortex (left/right) |  |
| 53/54 | Supramarginal gyrus (left/right) |  |
| 55/56 | Precuneus cortex (left/right) |  |
| 57/58 | Posterior cingulate cortex (left/right) |  |
| 59/60 | Isthmus cingulate cortex (left/right) | Not included |
| 61/62 | Lateral occipital cortex (left/right) | Occipital lobe |
| 63/64 | Cuneus cortex (left/right) |  |
| 65/66 | Pericalcarine cortex (left/right) |  |
| 67/68 | Lingual gyrus (left/right) |  |

**Supplemental Table 2. Detailed information about the network features used as input variables**

| Network attributes | Detail | Number of networks |
| --- | --- | --- |
| Global efficiency | Mean of efficiency calculated by 1/distance | 68 × 8 = 544 |
| Global efficiency (All) | Mean of global efficiency over all ROIs | 8 |
| Characteristic path length | Mean of path length (distance) | 68 × 8 = 544 |
| Characteristic path length (All) | Mean of the characteristic path length in all ROIs | 8 |
| Clustering coefficient (Binary) | Value explaining how much each node is clustered with other nodes. The binary distance is determined to be 1 if the edge exists; otherwise, it is 0. | 68 × 8 = 544 |
| Clustering coefficient (Binary, All) | Mean of clustering coefficient in all ROIs | 8 |
| Clustering coefficient (Weight) | Clustering coefficient calculated with weight (distance) | 68 × 8 = 544 |
| Clustering coefficient (Weight, All) | Mean of clustering coefficient in all ROIs | 8 |
| Modularity (Binary) | Degree of independence of each module after dividing the entire network into several groups(modules) | 8 |
| Modularity (Weight) | Degree of independence of each module after dividing the entire network into several groups (modules) | 8 |

**Supplemental Table 3. Correlation coefficient between top 20 selected features and the MoCA percentile score for the left stroke estimated group.**

| Network feature | Correlation coefficient |
| --- | --- |
| Global efficiency (Theta/ROI8) | 0.64 |
| Clustering coefficient (Weight/Theta/ROI3) | 0.43 |
| Clustering coefficient (Weight/Theta/ROI4) | 0.44 |
| Clustering coefficient (Weight/Delta/ROI9) | -0.46 |
| Clustering coefficient (Weight/Theta/ROI46) | 0.35 |
| Clustering coefficient (Binary/Theta/ROI44) | 0.33 |
| Global efficiency (Alpha2/ROI53) | 0.34 |
| Characteristic path length (Theta/ROI6) | -0.46 |
| Global efficiency (Alpha2/ROI61) | -0.31 |
| Clustering coefficient (Binary/Alpha2/ROI16) | 0.35 |
| Clustering coefficient (Weight/Theta/ROI60) | 0.3 |
| Global efficiency (Theta/ROI10) | 0.52 |
| Clustering coefficient (Binary/Beta2/ROI17) | -0.33 |
| Clustering coefficient (Binary/Alpha2/ROI12) | -0.32 |
| Clustering coefficient (Weight/Beta1/ROI19) | 0.33 |
| Clustering coefficient (Weight/Alpha2/ROI15) | 0.31 |
| Clustering coefficient (Weight/Theta/ROI44) | 0.36 |
| Characteristic path length (Alpha1/ROI40) | 0.36 |
| Clustering coefficient (Weight/Theta/ROI7) | 0.42 |
| Clustering coefficient (Weight/Beta1/ROI20) | 0.48 |

**Supplemental Table 4. Correlation coefficient between top 20 selected features and the MoCA percentile score for the right stroke estimated group.**

| Network feature | Correlation coefficient |
| --- | --- |
| Clustering coefficient (Weight/Alpha2/ROI5) | -0.49 |
| Clustering coefficient (Binary/Alpha2/ROI5) | -0.49 |
| Global efficiency (Beta2/ROI19) | -0.42 |
| Characteristic path length (Beta1/ROI11) | -0.34 |
| Characteristic path length (Beta1/ROI13) | -0.37 |
| Clustering coefficient (Weight/Theta/ROI8) | 0.32 |
| Characteristic path length (Gamma/ROI26) | 0.35 |
| Characteristic path length (Beta2/ROI14) | -0.4 |
| Clustering coefficient (Weight/Alpha1/ROI25) | 0.44 |
| Global efficiency (Delta/ROI61) | 0.37 |
| Modularity (Weight/Alpha2) | 0.43 |
| Clustering coefficient (Weight/Delta/ROI13) | 0.33 |
| Characteristic path length (Beta1/ROI53) | 0.45 |
| Global efficiency (Alpha2/ROI15) | -0.31 |
| Clustering coefficient (Weight/Beta2/ROI63) | -0.33 |
| Clustering coefficient (Binary/Beta2/ROI2) | -0.32 |
| Clustering coefficient (Weight/Beta1/ROI62) | 0.33 |
| Global efficiency (Beta2/ROI38) | 0.31 |
| Characteristic path length (Beta2/ROI29) | -0.35 |
| Clustering coefficient (Binary/Alpha1/ROI16) | 0.31 |
