# Supplementary figures and images for: Machine learning-based prediction of post-stroke cognitive status using electroencephalography-derived brain network attributes
Source: Front Aging Neurosci. 2023 Sep 28;15:1238274. doi: 10.3389/fnagi.2023.1238274 (PMC10568623; doi:10.3389/fnagi.2023.1238274)

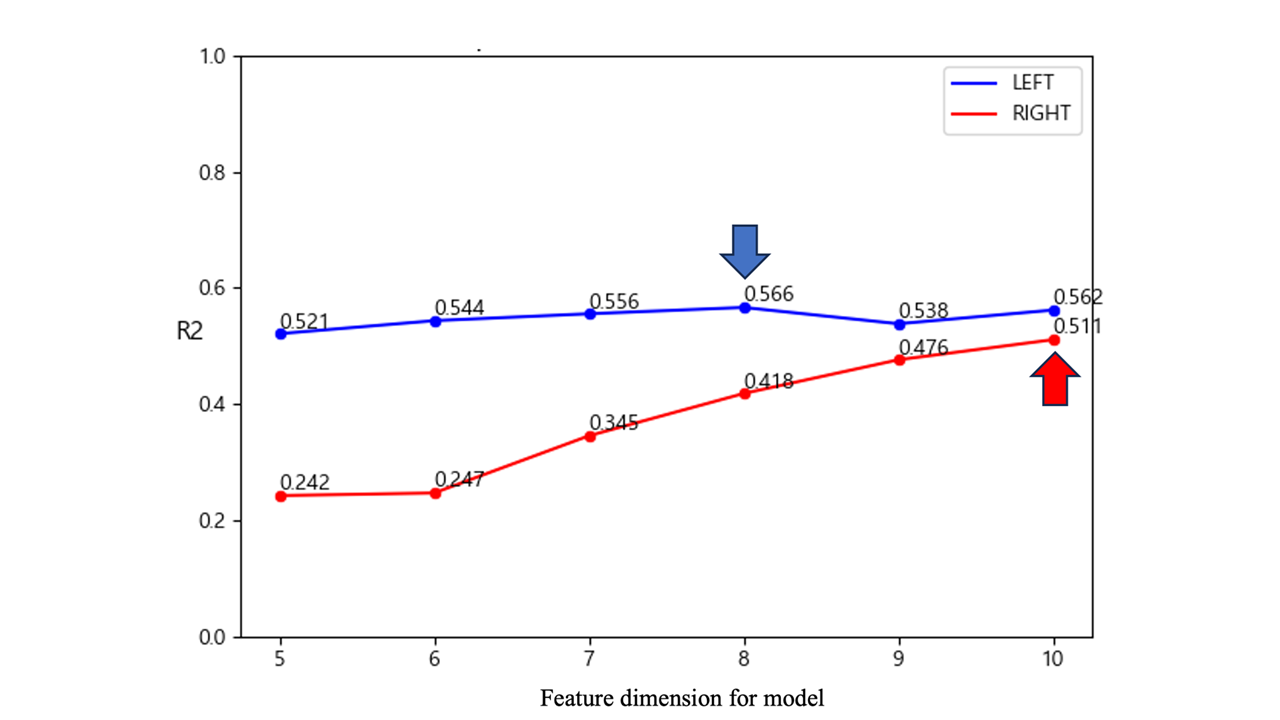

Supplement: Supplementary file 2 [file Image_1.PNG]

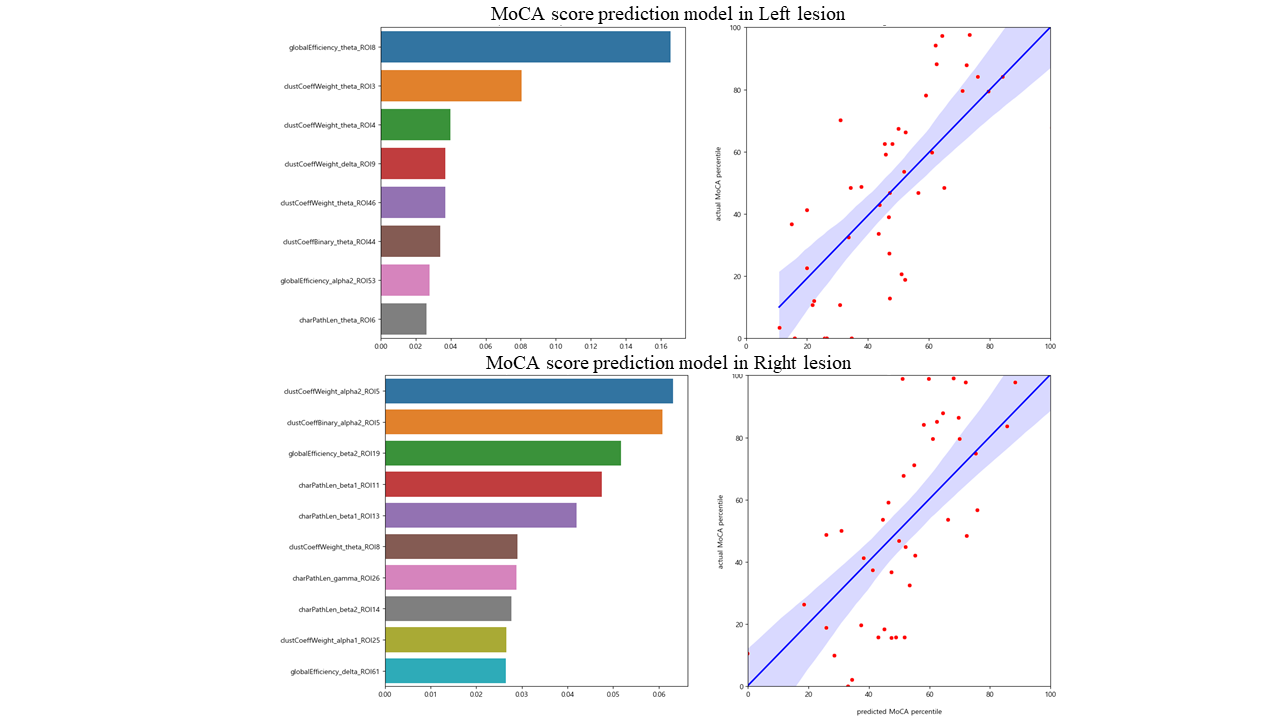

Supplement: Supplementary file 3 [file Image_2.PNG]

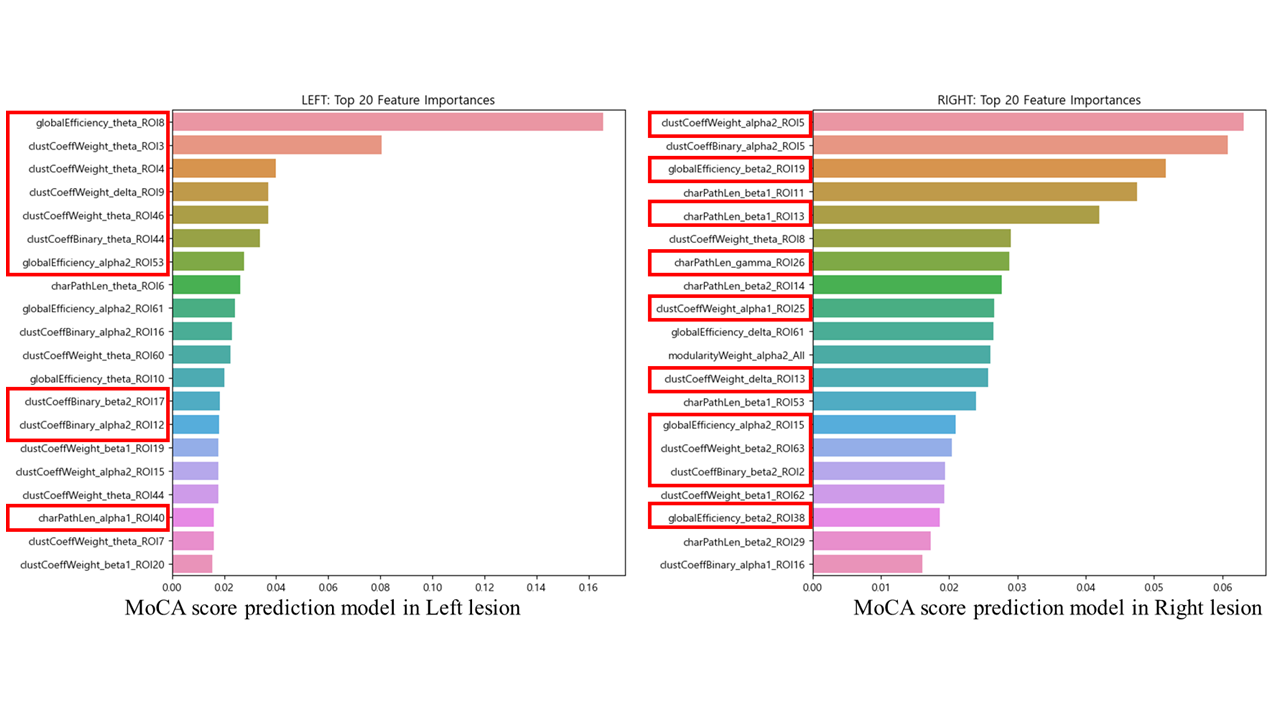

Supplement: Supplementary file 4 [file Image_3.PNG]
